# Learning curve for paramedic endotracheal intubation and complications

**DOI:** 10.1186/1865-1380-6-38

**Published:** 2013-10-17

**Authors:** Junko Toda, Alexis Akira Toda, Johji Arakawa

**Affiliations:** 1 Department of Anesthesiology, University of Tokyo, Tokyo, Japan; 2 Department of Economics, University of California San Diego, La Jolla, CA, USA; 3 Department of Anesthesiology, Japanese Red Cross Kitami Hospital, Kitami, Hokkaido, Japan

**Keywords:** Paramedic intubation, Learning curve, Complication, Success rate, Logit model

## Abstract

**Background:**

Pre-hospital laryngoscopic endotracheal intubation (ETI) is potentially a life-saving procedure but is a technique difficult to acquire. This study aimed to obtain a recommendation for the number of times ETI should be practiced by constructing the learning curve for endotracheal intubation by paramedics, as well as to report the change in the frequency of complications possibly associated with intubation over the training period.

**Methods:**

Under training conditions, 32 paramedics performed a total of 1,045 ETIs in an operating room. Trainees performed ETIs until they succeeded in 30 cases. For each patient, the number of laryngoscopic maneuvers and any complications potentially associated with ETI were recorded. We built a generalized logistic model to construct the learning curve for ETI and the frequency of complications.

**Results:**

During the training on the first 30 patients the rate of ETI success at the first attempt improved from 71% to 87%, but there was little improvement during the first 13 cases. The frequency of complications decreased from 53% to 31%. More laryngoscopic maneuvers and longer operation time increased complications.

**Conclusions:**

It seems that 30 live experiences of performing an ETI is sufficient for obtaining a 90% ETI success rate, but there seems to be little benefit with fewer than 13 experiences. The frequency of complications remained at a high level even after the training. It is desirable to conduct a more detailed and rigorous evaluation of the benefit of pre-hospital ETI by controlling for the skill level of paramedics.

## Background

In Japan, until 2004 only medical doctors were legally allowed to perform an endotracheal intubation on patients. In 2004, the Japanese Ministry of Health, Labour and Welfare legalized pre-hospital endotracheal intubation (ETI) by paramedics who have successfully completed a standardized training program, which consists of learning the theoretical aspects of ETI from a lecture and a video, practicing on a mannequin and 30 live experiences of intubation in an operating room.

The objective of this study was to assess the efficacy of the training program from the following two points of view: (i) How much does the success rate of ETI improve over the course of the 30 live experiences? (ii) How much does the frequency of complications possibly associated with ETI decrease? These questions are important for the following reasons. First, the benefit of pre-hospital ETI over bag-valve-mask ventilation remains controversial [1-6]. For pre-hospital ETI to have any benefit, the performer of ETI must possess a minimum acceptable skill level. Therefore the heterogeneity in the skill levels of paramedics across studies might be a source of controversy regarding the benefit of pre-hospital ETI over bag-valve-mask ventilation. Second, knowing the learning curve of ETI is helpful in designing training programs because opportunities for practicing ETI are limited, especially for paramedics. Third, although the learning curve for endotracheal intubation has been studied [7-11], how fast complications associated with endotracheal intubation diminish seems to be an untouched subject. We also study the factors associated with complications.

## Methods

### Study population

Following institutional review board approval (Japanese Red Cross Kitami Hospital, Kitami, Hokkaido, Japan), a total of 32 paramedics were trained in laryngoscopic endotracheal intubation (ETI) from January 2005 to December 2011. None of the trainees had prior experience of live ETI. All trainees were formally trained in the theoretical aspects of ETI through attending a standardized lecture, watching a video and then practicing on a mannequin before participating in the study. This training was given by one instructor (Arakawa). Healthy surgical patients who required an endotracheal tube as part of their anesthetic management were recruited to the study and provided written informed consent. The inclusion criteria were: (i) age 20 years or older, (ii) American Society of Anesthesiologists (ASA) physical status class I or II and (iii) no evidence of a potentially difficult airway. Under a standardized anesthesia technique with muscle relaxation (5 mg/kg thiamylal sodium and 0.07 to 0.1 mg/kg vecuronium), these patients underwent ETI by the trainees, with an attending anesthesiologist present and providing ongoing supervision. For each patient the trainee was given two opportunities to perform the laryngoscopic maneuver. If the trainee failed to complete ETI after two attempts (an attempt is defined by a laryngoscopic maneuver, that is, the insertion of the laryngoscope blade into the mouth), the attending anesthesiologist took over. The attending anesthesiologist recorded the number of intubation attempts (one, two or three if the attending anesthesiologist took over) and evaluated any complications possibly associated with intubation right after the completion of ETI as well as at the post-operative visit. A complication was defined by one (or more) of the following symptoms: hoarseness, sore throat, lip laceration, oral bleeding, gingival bleeding, lip bleeding, pharyngeal bleeding, tongue laceration, dental damage, lip swelling or tongue bleeding. Some symptoms may have been caused not by the intubation but the subsequent operation, but we decided to include as many potential complications as possible to produce a conservative estimate. Each trainee continued training until completed 30 successful ETIs had been completed in total.

### Evaluation of ETI

We defined the completion of ETI by a proper placement of the endotracheal tube (auscultation of stomach and both lungs and end-tidal carbon dioxide measurement) after one or two laryngoscopic maneuvers. For the purpose of statistical analysis we defined 'success at first attempt’ and 'success at second attempt’ by the completion of ETI after one and two laryngoscopic maneuvers, respectively. We distinguished these two events because they are not statistically independent since failure in the first attempt might be associated with problems with the patient’s airway or the trainee might obtain useful information (such as the anatomical structure of the patient’s airway) during the first attempt.

### Statistical analysis

We built a generalized logistic model similar to, but more general and flexible than, that of Mulcaster *et al.*[9] to construct the learning curve for successful ETI as well as complications. More precisely, the model for the ETI success rate is:

(1) $$\mathrm{Pr}(\text{success}|x)=P_{i}+\frac{P_{f}-P_{i}}{1+\exp(-V(x-T))},$$

where *x* is the number of experiences (x=1,2,…,30), *P*_*i*_ and *P*_*f*_ are the initial and final success rates corresponding to *x* → ±*∞*, *V* is the learning speed and *T* is the number of experiences at which the success rate improves fastest. We define a successful intubation as completing ETI at the first attempt. In Model 1 only experience *x* is a relevant explanatory variable because the age and sex of the patient were found to be insignificant in a preliminary analysis. We did not include the number of elapsed days since the first day of training as a regressor because it has been found to be insignificant [10]. The usual logistic model with no initial or final skill level is a special case of Model 1 by setting *P*_*i*_ = 0 and *P*_*f*_ = 1. We estimated the model parameters *P*_*i*_,*P*_*f*_,*V* and *T* using the maximum likelihood and obtained the 95% confidence interval of each parameter as well as the success rate by bootstrapping 1,000 times [12].

We also applied a generalized logistic model similar to Model 1 to analyze the probability of complications associated with ETI. The full model is:

(2) $$\mathrm{Pr}(\text{complication}|x)=P_{i}+\frac{P_{f}-P_{i}}{1+\exp(-\beta^{\prime }x)},$$

where *P*_*i*_ and *P*_*f*_ are the initial and final complication rates, ***β*** is the vector of coefficients and **x** is the vector of explanatory variables that includes a constant, the experience, the operation time (in minutes), dummy variables for failure in the first and second intubation attempts for each patient, the patient’s age and sex, and whether a nasogastric tube was inserted. As before we estimated Model 2 using the maximum likelihood and obtained the 95% confidence interval of each parameter as well as the complication rate by bootstrapping 1,000 times. Since we failed to reject the simple logistic model with no starting skill level (*P*_*i*_ = 1) and no final skill level (*P*_*f*_ = 0) (*P* = 1.00), we re-estimated the model by setting *P*_*i*_ = 1 and *P*_*f*_ = 0.

All statistical analyses were conducted using Matlab v8.0.0 (The MathWorks, Inc., Natick, MA, USA). Estimation by maximum likelihood was performed using the fminsearch command and the 95% confidence intervals were obtained by the bootci command. We applied the likelihood ratio test [13] for all hypothesis testing.

## Results

Overall, 32 paramedics attempted 1,049 laryngoscopic endotracheal intubations. Four cases were aborted because of the failure in visualizing the vocal cords in one patient, tooth mobility in another and dental damage during the bag-valve-mask ventilation in two others. Of the remaining 1,045 cases, for each trainee we used only the data corresponding to the first 30 patients to avoid introducing survival bias. Therefore the total number of observations used in the data analysis was 32×30=960.

### ETI success

**Figure 1:**
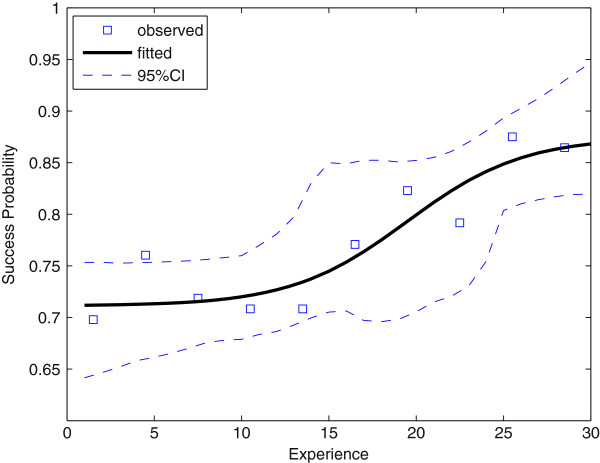
**Observed success frequency of endotracheal intubation and estimated probability from Model 1**. The dashed curves indicate the 95% confidence interval.

To visualize the learning curve, in Figure 1 we plot the observed ETI success frequency computed over ten intervals with equal length (experience from 1 to 3, 4 to 6 and so on) as well as the estimated probability (see the Appendix for details). Table 1 presents the parameter estimates and confidence intervals.

**Table 1:** **Estimation result of Model 1**

**Parameter**	**Estimate**	**95% CI**^**a**^
*P*_*i*_	0.71	[0.46, 0.75]
*P*_*f*_	0.87	[0.82, 1.0]
*V*	0.30	[0.040, 22]
*T*	19	[14, 29]

^a^ 95% confidence interval obtained by bootstrapping 1,000 times.

According to Figure 1, the fitted probability closely tracks the observed frequency. In particular, the fitted probability shows no substantial improvement before 15 experiences but sharply increases between 15 and 25 experiences. The reason why the 95% confidence intervals are wider in this region is because the fitted success rate (right-hand side of Equation 1) is most sensitive to the learning speed *V* in this region, hence the confidence intervals widen because of the sampling error in *V*.

To test that there is initially no substantial improvement in the ETI success rate, we assume there is no learning effect up to some threshold for experiences and estimate Model 1 with the threshold with highest log-likelihood. More precisely, the new model is that Equation 1 holds for *x* > *k* but Pr(success|*x*) = constant for *x* ≤ *k*, where *k* is the threshold. The resulting threshold was 13 and the null hypothesis 'no substantial learning up to some threshold of experiences’ was not rejected by the likelihood ratio test (*P* = 0.44, one degree of freedom).

Overall the success rate improved from 71% to 87% after training on 30 patients. In fact, the null hypothesis of no learning (*V* = 0 in Model 1) was rejected by the likelihood ratio test (*P* = 0.0019). Even with no training the success rate was positive (*P*_*i*_ = 0 in Model 1 was rejected, *P* = 0.0016), but the success rate did not necessarily plateau at a level below 100% (*P*_*f*_ = 1 in Model 1 was not rejected, *P* = 0.65).

To evaluate the model fit, we divided experience into 30, 15 and 10 categories corresponding to intervals with length 1, 2 and 3 and performed the likelihood ratio test for goodness-of-fit (to this end, we compared the baseline Model 1 to a multinomial distribution; see the Appendix for more details). The result was *P* = 0.38,0.42 and 0.62 in each case, suggesting that the current generalized logistic model fits well to the data.

Although our data involved 30 experiences or less for each trainee, we can compute how many experiences are necessary to achieve a prescribed success rate if we believe that the model can be extrapolated. To this end, we used the baseline Model 1 with final probability *P*_*f*_ = 1 (which was not rejected) and found the number of experiences *x* that gave the prescribed success rate. The confidence intervals were obtained by bootstrapping as before. The result was *x* = 31.5 (95% CI: [27.6, 54.3]) for 90% success rate and *x* = 38.6 (95% CI: [31.2, 76.9]) for 95% success rate.

To evaluate the robustness of Model 1, we performed a number of robustness checks. Although the learning curve in Figure 1 is S-shaped, Gallistel *et al.*[14] report that the negatively accelerated, gradually increasing learning curve is an artifact of group averaging. To deal with the possibility that individual learning curves deviate from the average learning curve, we estimated Model 1 by allowing one of the parameters *V*, *T*, or both to vary across paramedics. However, the baseline Model 1 without individual fixed effects was not rejected by the likelihood ratio test (*P* = 1.00 in all three cases). Thus the learning curve for ETI did not appear to vary across individuals.

In Model 1 we assumed that experience *x* enters linearly in the logistic function. To deal with potential nonlinearity, we added a quadratic term *a*(*V*(*x*-*T*))^2^ into the logistic function in Equation 1, where *a* is a coefficient. However, the likelihood ratio test failed to reject the baseline Model 1, which corresponds to *a* = 0 (*P* = 0.86). Therefore, the baseline Model 1 seemed appropriate.

### Complications

Table 2 shows the prevalence of complications possibly associated with ETI (some patients experienced two or more). Except for three cases of dental damage, the complications were minor^a^.

**Table 2:** The prevalence of complications possibly associated with laryngoscopic endotracheal intubation

**Complication**	**Number of cases**	**Percentage in sample**
Hoarseness	307	29%
Sore throat	189	18%
Lip laceration	15	1.4%
Oral bleeding	13	1.2%
Gingival bleeding	5	0.48%
Lip bleeding	5	0.48%
Pharyngeal bleeding	4	0.38%
Tongue laceration	4	0.38%
Dental damage	3	0.29%
Lip swelling	2	0.19%
Tongue bleeding	1	0.1%

**Figure 2:**
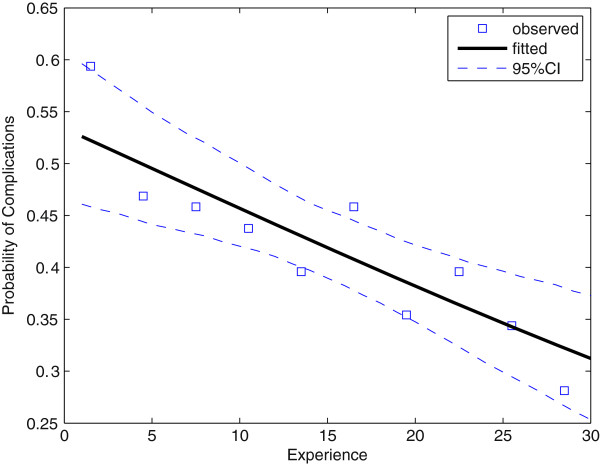
**Observed frequency of complications and estimated probability from Model2**.The dashed curves indicate the 95% confidence interval.

Figure 2 plots the observed frequency of complications as well as the estimated probability. Table 3 presents the parameter estimates and confidence intervals. Overall the complication rate decreased from 53% to 31% after training on 30 patients. A patient’s age and sex and whether a nasogastric tube was inserted were jointly insignificant (*P* = 0.21). More experience decreased the complications (*P* = 0.0055) and longer operation time and one or two failures in intubation attemps increased the complications (*P* = 0.016,0.0060, respectively), but there was no difference in complications between one and two failures (*P* = 0.75). This was probably because if the trainee failed twice, the attending anesthesiologist took over, whose laryngoscopic maneuver is minimally invasive.

**Table 3:** **Estimation result of Model2**

**Variable**	**Estimate**	**95% CI**^**a**^
Experience	0.031	[0.016, 0.047]
Operation time	-0.0026	[-0.004, -0.0007]
One failure	-0.73	[-1.2, -0.027]
Two failures	-0.86	[-1.5, -0.02]

^a^ 95% confidence interval obtained by bootstrapping 1,000 times.

## Discussion

Considering the limited opportunities for paramedics to practice ETI, the learning curve in Figure 1 suggests that requiring 30 live experiences seems to be reasonable since after 30 live experiences the success rate was 87% (95% CI: 82 to 94%). However, whether paramedics should be trained in ETI in the first place or whether paramedics should perform pre-hospital ETI is another issue. Although there is some evidence that endotracheal intubation in the field by paramedics improves survival and functional outcome in patients with head injury [1,6], other studies report negative results [2-5]. A more detailed and rigorous evaluation of the benefit of pre-hospital ETI is desirable. In that case it will be important to control for the skill level of paramedics participating in the study, since we found that there is no significant learning effect up to 13 live experiences.

The National Standard Paramedic Curriculum in the US recommends that paramedic students perform at least five live endotracheal intubations [10], but the learning curve in Figure 1 suggests that five experiences are insufficient since we found that there is no significant learning up to 13 experiences and learning is fastest at around 19 experiences. Since the simulation of ETI with a mannequin is reported to be effective [15], trainees with limited training opportunities (especially paramedics) should thoroughly practice with mannequins before proceeding to live ETI to get the most out of those opportunities.

Although complications associated with ETI are well known [16,17], there are hardly any reports on the dependence of the complication rate on the experience of the performer. According to Figure 2 the frequency is quite high among novices, but quickly diminishes with acquired experience. The vast majority of complications are minor, among which hoarseness and sore throat are the most common. Not all of these cases were caused by intubation per se, since according to Conway *et al.*[18] sore throat occurs in about 10% of all post-operative patients (excluding those who underwent pharyngeal and laryngeal operations) who were not intubated. However, the curve in Figure 2 does provide an upper bound (conservative estimate) of complications caused by intubation. Table 4 summarizes the baseline and final probabilities (probabilities before and after training) and the threshold number of experiences for the improvement of performance.

**Table 4:** Summary

	**ETI success**	**Complication**
Baseline probability (before training)	71%	53%
Final probability (after training)	87%	31%
Threshold for improvement	13	0

The learning curve for anesthetic procedures has been documented by a number of researchers using simple visualization [19], the cusum method [7,8,20,21] and logistic regression [9-11], among others. Simple visualization such as the observed frequency in Figures 1 and 2 is always helpful to avoid specifying a highly inappropriate model. Without visualization we would not have modeled the initial and final success rate as in Model 1. However, currently there is no universally accepted method for modeling the learning curve (see [22,23] for systematic reviews). Statistical methods for modeling the learning curve ideally should aim to estimate three parameters: rate of learning, baseline (starting) skill level and final skill level (asymptote) [23]. Our proposed Model 1, of course, passes these criteria.

The cusum method [24], despite its wide use, is problematic for modeling the learning curve. First, the cusum method was originally developed for quality control to detect a process out of control. Since by design the cusum method can only be applied to a process with a linear trend, it might be useful for detecting the emergence of a learning effect (or detecting a trainee who is less proficient) but is not suitable for modeling the entire learning curve where the success rate changes nonlinearly over time. Second, explanatory variables other than time cannot be included in the cusum method. Third, the cusum method is unable to estimate the rate of learning, the baseline skill level or the final skill level.

The generalized logistic model we applied to construct the learning curve is flexible enough to fit the data well but specialized enough to be able to estimate the parameter of interest. We hope that researchers interested in the learning curve will broaden their analytical tools.

### Limitations

This study has a number of limitations. First, because the study was carried out at a single institution, the particular learning curve we obtained is strongly influenced by the teaching quality of this particular institution. Second, in our study we included only healthy surgical patients with no sign of an obviously difficult airway. Thus it is unclear whether our results will remain valid for the whole population, although our learning curve for ETI can be interpreted as the upper bound (optimistic estimate) of the true learning curve with the general population. Third, as the paramedics were trained only up to 30 experiences, the extrapolation of the learning curve beyond 30 cases should be taken with caution. Finally, and perhaps most importantly, the outcome of this study (success/failure of intubation under muscle relaxation in the operating room) is necessarily a short-term goal. The long-term goal is whether paramedic intubation is beneficial to actual emergency patients, but our study does not address this question. However, our study does point out the importance of controlling the skill level of paramedics when evaluating the benefit of paramedic pre-hospital intubation.

## Conclusions

Any training program should be evaluated for efficacy. We constructed the learning curve for paramedic intubation and found that 30 live experiences of laryngoscopic endotracheal intubation seems to be sufficient for obtaining a 90% success rate in an operating room, but there seems to be little benefit with fewer than 13 experiences. The frequency of complications remains at a high level even after training. It is desirable to conduct a more detailed and rigorous assessment of the benefit of pre-hospital intubation that controls for the skill level of paramedics.

## Endnote

^a^ An anonymous reviewer suggested reporting the baseline complications of the anesthesiologists that usually perform intubation at this institution for comparison to the paramedics. Unfortunately, no data were available since minor complications are often left unreported by anesthesiologists.

## Appendix

### Generalized logistic model

Suppose an outcome *y* is binary, say *y* = 0,1 or 'success’ and 'failure’, and we are interested in the relation between some explanatory variables **x** and the probability of the outcome, Pr(*y* = 1|**x**). A typical way to model this situation is the linear logistic model:

(3) $$\mathrm{Pr}(y=1|x)=\frac{1}{1+\exp(-\beta^{\prime }x)},$$

where ***β*** is the vector of coefficients of the regressors. The simple logistic Model 3 may be useful most of the time, but it has the disadvantage that the initial and final probability of 'success’ is necessarily 0 and 1. To see this, suppose that the regressors include time and let time be very small or very large. Then the right-hand side of 3 tends to 0 or 1. For this reason, we use the more general model:

(4) $$\mathrm{Pr}(y=1|x)=P_{i}+\frac{P_{f}-P_{i}}{1+\exp(-\beta^{\prime }x)},$$

where *P*_*i*_ and *P*_*f*_ are the initial and final probabilities of 'success’. Model 4 includes the simple logistic Model 3 by setting *P*_*i*_ = 0 and *P*_*f*_ = 1, which Mulcaster *et al.*[9] use to model successful ETI.

### Estimation

Because Model 4 is fully parametric, the most natural way to estimate it is by maximum likelihood. Let ${\left(y_{n},x_{n}\right)}_{n=1}^{N}$ be the data. Then the log-likelihood function is given by:

(5) $$\begin{array}{lll} \log L(P_{i},P_{f},\beta )\;= & \overset{N}{\underset{n=1}{\sum }}\;\left[\;y_{n}\log\;\left(P_{i}\;+\;\frac{P_{f}-P_{i}}{1+\exp(-\beta^{\prime }x_{n})}\right)\right)\; & \;\\ & \left(+(1\;-\;y_{n})\;\log\;\left(\;1\;-\;P_{i}-\;\frac{P_{f}-P_{i}}{1\;+\;\exp(-\beta^{\prime }x_{n})}\;\right)\;\right]\;.\; \end{array}$$

The maximum likelihood estimator $\left(\hat{P_{i}},\hat{P_{f}},\hat{\beta }\right)$ can be obtained by maximizing the log-likelihood function 5 subject to 0 ≤ *P*_*i*_,*P*_*f*_ ≤ 1 using optimization routines. For instance, in this paper we use the fminsearch command in Matlab v8.0.0 (The MathWorks, Inc., Natick, MA, USA).

### Confidence intervals

Under general conditions the maximum likelihood estimator is consistent and asymptotically normal. Therefore in large samples the standard errors and confidence intervals of parameters can be obtained using the asymptotic variance. This approach has the disadvantage that the approximation may not be good in small samples and we need to compute higher-order derivatives of the log-likelihood function. An alternative is to use the bootstrap [12]. For each bootstrap repetition b=1,2,…,B (say *B* = 1,000), we construct a bootstrap sample ${\left(y_{n}^{b},x_{n}^{b}\right)}_{n=1}^{N}$ by resampling (with replacement) from the original sample ${\left(y_{n},x_{n}\right)}_{n=1}^{N}$ with probability 1/*N* for each observation. Then we estimate the model by maximum likelihood using each bootstrap sample and obtain the 100(1-*α*)% confidence interval of any parameter of interest by reporting the *α*/2 and 1-*α*/2 quantiles of the bootstrap estimates corresponding to b=1,2,…,B. In this paper we obtain the 95% confidence intervals using the bootci command in Matlab.

### Model selection and goodness-of-fit

Model 4 actually contains many models, selected by choosing a different set of explanatory variables **x** or restricting the initial and final probabilities *P*_*i*_ and *P*_*f*_. How should we choose from different models? If one model is nested within another (that is, one model is a special case of another), we can use the likelihood ratio test [13]. Suppose there are two models, 1 and 2, where Model 1 is a special case of Model 2. Let *L*_1_ and *L*_2_ be the likelihood of each model obtained by maximum likelihood estimation. Then the logarithm of the likelihood ratio statistic 2(log*L*_2_- log*L*_1_) is asymptotically chi-squared distributed with degrees of freedom *k*_2_-*k*_1_, where *k*_1_ and *k*_2_ are the number of parameters in Models 1 and 2. This is the likelihood ratio test. To compare models that are not necessarily nested, we can use either the Akaike Information Criterion [25] or the Bayesian Information Criterion [26], but for the data used in this paper the likelihood ratio test suffices.

To test the model fit, suppose that the value of the explanatory variables **x** falls in one of *J* categories. Then Model 4 is a special case of the model in which the outcome *y* comes from a binomial distribution with success rate *p*_*j*_, where j=1,2,…,J. Thus evaluating the model fit reduces to the comparison between two nested models, hence we can apply the likelihood ratio test. More precisely, let *N*_*j*_ be the number of observations in category *j*, of which there are *N*_*j*_ 'successes’. Then the maximum likelihood estimate of the binomial model is ${\hat{p}}_{j}=n_{j}/N_{j}$, with log-likelihood:

(6) $$\overset{J}{\underset{j=1}{\sum }}\left[n_{j}\log{\hat{p}}_{j}+(N_{j}-n_{j})\log(1-{\hat{p}}_{j})\right].$$

On the other hand, the fitted probability of 'success’ in category *j* using Model 4 is given by:

$${\hat{q}}_{j}=\frac{1}{N_{j}}\underset{x_{n}\in j}{\sum }\left[\hat{P_{i}}+\frac{\hat{P_{f}}-\hat{P_{i}}}{1+\exp(-{\hat{\beta }}^{\prime }x_{n})}\right].$$

Then the log-likelihood of Model 4 with *J* categories is given by Equation 6 with ${\hat{p}}_{j}$ replaced by ${\hat{q}}_{j}$. Having obtained two log-likelihoods, we can perform the likelihood ratio test. For more information see Chapter 5 of Hosmer and Lemeshow [27].

## Abbreviations

ETI: Laryngoscopic endotracheal intubation.

## Competing interests

The authors declare that they have no competing interests.

## Authors’ contributions

JT participated in the design and coordination of the study and drafted the manuscript. AAT performed the statistical analysis and drafted the manuscript. JA conceived and designed the study and collected data. All authors read and approved the final manuscript.

## References

[B1] Winchell RJ Hoyt DB Endotracheal intubation in the field improves survival in patients with severe head injury Arch Surg 1997 6 6 592 597 10.1001/archsurg.1997.01430300034007 9197850

[B2] Katz SH Falk JL Misplaced endotracheal tubes by paramedics in an urban emergency medical services system Ann Emerg Med 2001 6 32 37 10.1067/mem.2001.112098 11145768

[B3] Stockinger ZT McSwain NEJ Prehospital endotracheal intubation for trauma does not improve survival over bag-valve-mask ventilation J Trauma Acute Care Surg 2004 6 3 531 536 10.1097/01.TA.0000111755.94642.29 15128123

[B4] Davis DP Peay J Sise MJ Vilke GM Kennedy F Eastman AB Velky T Hoyt DB The impact of prehospital endotracheal intubation on outcome in moderate to severe traumatic brain injury J Trauma Acute Care Surg 2005 6 5 933 939 10.1097/01.TA.0000162731.53812.58 15920406

[B5] Lecky F Bryden D Little R Moulton C Emergency intubation for acutely ill and injured patients Cochrane Database Syst Rev 2008 6 CD001429 18425873 10.1002/14651858.CD001429.pub2 PMC7045728

[B6] Bernard SA Nguyen V Cameron P Masci K Fitzgerald M Cooper DJ Walker T Myles P Murray L Taylor DM Smith K Patrick I Edington J Bacon A Rosenfeld JV Judson R Prehospital rapid sequence intubation improves functional outcome for patients with severe traumatic brain injury: a randomized controlled trial Ann Surg 2010 6 6 959 965 10.1097/SLA.0b013e3181efc15f 21107105

[B7] Konrad C Schüpfer G Wietlisbach M Gerber H Learning manual skills in anesthesiology: is there a recommended number of cases for anesthetic procedures? Anesth Analg 1998 6 3 635 639 9495429 10.1097/00000539-199803000-00037

[B8] de Oliveira Filho GR The construction of learning curves for basic skills in anesthetic procedures: an application for the cumulative sum method Anesth Analg 2002 6 2 411 416 12145063 10.1097/00000539-200208000-00033

[B9] Mulcaster JT Mills J Hung OR MacQuarrie K Law JA Pytka S Imrie D Field C Laryngoscopic intubation: learning and performance Anesthesiology 2003 6 23 27 10.1097/00000542-200301000-00007 12502974

[B10] Wang HE Seitz SR Hostler D Yealy DM Defining the 'learning curve’ for paramedic student endotracheal intubation Prehosp Emerg Care 2005 6 2 156 162 10.1080/10903120590924645 16036839

[B11] Warner KJ Carlbom D Cooke C Bulger EM Copass MK Sharar SR Paramedic training for proficient prehospital endotracheal intubation Prehosp Emerg Care 2010 6 103 108 10.3109/10903120903144858 19947874

[B12] Efron B Bootstrap methods: another look at the jackknife Ann Stat 1979 6 1 26 10.1214/aos/1176344552

[B13] Wilks SS The large-sample distribution of the likelihood ratio for testing composite hypotheses Ann Math Stat 1938 6 60 62 10.1214/aoms/1177732360

[B14] Gallistel CR Fairhurst S Balsam P The learning curve: implications of a quantitative analysis Proc Natl Acad Sci 2004 6 36 13124 13131 10.1073/pnas.0404965101 15331782 PMC516535

[B15] Hall RE Plant JR Bands CJ Wall AR Kang J Hall CA Human patient simulation is effective for teaching paramedic students endotracheal intubation Acad Emerg Med 2005 6 9 850 855 10.1111/j.1553-2712.2005.tb00961.x 16141019

[B16] Blanc VF Tremblay N The complications of tracheal intubation: a new classification with a review of the literature Anesth Analg 1974 6 2 202 213 4593090

[B17] Mort TC Emergency tracheal intubation: complications associated with repeated laryngoscopic attempts Anesth Analg 2004 6 2 607 613 15271750 10.1213/01.ANE.0000122825.04923.15

[B18] Conway CM Miller JS Sugden FLH Sore throat after anaesthesia Brit J Anaesth 1960 6 219 223 10.1093/bja/32.5.219 13811654

[B19] Kopacz DJ Neal JM Pollock JE The regional anesthesia 'learning curve’: what is the minimum number of epidural and spinal blocks to reach consistency? Reg Anesth 1996 6 3 182 190 8744658

[B20] Kestin IG A statistical approach to measuring the competence of anaesthetic trainees at practical procedures Brit J Anaesth 1995 6 6 805 809 10.1093/bja/75.6.805 8672339

[B21] de OliveiraFilho GR da Conceição DB Garzel IS Pavei P Ceccon MS Helayel P E Learning curves and mathematical models for interventional ultrasound basic skills Anesth Analg 2008 6 2 568 573 10.1213/ane.0b013e3181605412 18227318

[B22] Ramsey CR Grant AM Wallace SA Garthwaite PH Monk AF Russell IT Assessing the learning curve effect in health technologies: a systematic review Int J Technol Assess Health Care 2000 6 4 1095 1108 10.1017/S0266462300103149 11155830

[B23] Ramsey CR Wallace SA Garthwaite PH Monk AF Russell IT Grant AM Assessing the learning curve effect in health technologies: lessons from the nonclinical literature Int J Technol Assess Health Care 2002 6 1 10 11987432

[B24] Biau DJ Resche-Rigon M Godiris-Petit G Nizard RS Porcher R Quality control of surgical and interventional procedures: a review of the CUSUM Qual Saf Health Care 2007 6 203 207 10.1136/qshc.2006.020776 17545347 PMC2464981

[B25] Akaike H A new look at the statistical model identification IEEE Trans Automatic Control 1974 6 6 716 723

[B26] Schwarz G Estimating the dimension of a model Ann Stat 1978 6 2 461 464 10.1214/aos/1176344136

[B27] Hosmer DW Lemeshow S Applied Logistic Regression. 2nd edition. In Wiley Series in Probability and Statistics 2000 New York: John Wiley & Sons

